# Cecal microbiota of broilers responds similarly to black soldier fly larvae fat and conventional dietary fat sources

**DOI:** 10.1371/journal.pone.0336523

**Published:** 2025-11-25

**Authors:** Muhammad Rumman Aslam, Bartosz Kierończyk, Piotr Szymkowiak, Liliana Ciesielska, Mateusz Rawski, Robert Mikuła, Damian Józefiak

**Affiliations:** 1 Department of Animal Nutrition, Faculty of Veterinary Medicine and Animal Science, Poznań University of Life Sciences, Poland; 2 Laboratory of Inland Fisheries and Aquaculture, Department of Zoology, Faculty of Veterinary Medicine and Animal Science, Poznań University of Life Sciences, Poland; Universitas Sebelas Maret, INDONESIA

## Abstract

This study aimed to compare the effects of black soldier fly (*Hermetia illucens*) larvae (BSFL) fat with those of dietary fats commonly used in broiler chicken nutrition on gut pH and cecal microbiome. A total of 800 one-day-old male Ross 308 chicks were randomly assigned to eight dietary groups, with each group consisting of 10 replicate pens of 10 birds each fed for 35 days. The study design was as follows: the basal diet was enriched with various dietary fats as the sole source of fat, including BSFL fat (as the reference group), soybean oil (SO), rapeseed oil (RO), palm oil (PO), palm kernel fatty acid distillate (PKFD), poultry fat (PF), pig lard (PL), and beef tallow (BT). At the end of the experiment (35 days), the digesta from the crop, gizzard, jejunum, and ceca were sampled for further analyses, including pH determination and next-generation sequencing (NGS). Compared with PKFD, PF, and BT, BSFL significantly reduced the crop pH (P = 0.005). Additionally, BSFL increased the gizzard pH (P = 0.006) relative to PKFD. No differences in alpha diversity were detected among the diets; however, beta diversity differed significantly between the BSFL and PKFD groups (P = 0.034). BSFL fat was associated with a significant reduction in the abundances of Proteobacteria (P = 0.011), Enterobacteriaceae (P = 0.009), and *Escherichia-Shigella* (P = 0.009) compared with PKFD fat. LEfSe analysis revealed the following microbial markers responsive to BSFL treatment: total bacteria (P < 0.001), Rikenellaceae (P = 0.025), Peptococcaceae [uncultured genus] (P = 0.003), Rhodospirillales (P = 0.048), *Alistipes* (P = 0.025), the *Eubacterium coprostanoligenes* group (P = 0.018), the *Clostridia vadin* BB60 group (P = 0.032), and *Alistipes* sp. (P = 0.023). These findings suggest that BSFL positively affects the pH in the upper part of a bird’s gut compared with selected animal fats. Furthermore, BSFL enriched beneficial bacteria while inhibiting opportunistic pathogens in the cecal environment of broiler chickens.

## Introduction

Dietary fats, as concentrated sources of energy in poultry diets, increase nutrient absorption and feed palatability [1]. Nevertheless, plant oils and animal fats influence the gut environment. Unsaturated fatty acid (UFA) sources, such as soybean or rapeseed oils, improve cecal microbial diversity and reduce abdominal fat deposition compared to saturated fatty acid (SFA) sources, such as beef tallow [2]. Unlike UFAs, most SFAs are esterified at the sn-1 and sn-3 positions on the glycerol backbone, which reduces their digestibility and increases digesta viscosity [3]. This leads to the fermentation of undigested proteins, producing harmful biogenic amines and phenolic compounds [4]. Black soldier fly larval (BSFL) fat is a sustainable alternative because of its relatively low environmental impact and nutritional profile, which is dominated by medium-chain fatty acids (MCFAs), particularly lauric acid (LA) [5], and high metabolizable energy [6]. LA is known to have antimicrobial properties, enhancing immunity, supporting gut integrity by promoting the renewal and repair of intestinal epithelial cells, and reducing inflammation [7]. Previous research has indicated that BSFL fat has positive effects on the cecal microbiota and improves physiological and immunological responses, making it a promising fat source in broiler diets [8,9].

Feed materials, including fats, influence the gastrointestinal tract (GIT) environment by altering the pH and microbial composition [10]. Maintaining the optimal GIT digesta pH is essential for nutrient utilization and digestive health in broilers [11]. UFA-rich diets are more digestible than those rich in SFAs, reducing free fatty acid (FFA) concentrations in the lower GIT and stabilizing the pH [12]. Moreover, Józefiak et al. [13] reported that animal fats (lard and beef tallow) reduced crop pH and increased LA and volatile fatty acid (VFA) levels but had no effect on pH in the ileum or ceca. However, palm kernel fatty acid distillates increased the pH in the ileum compared with beef tallow and soybean oil while reducing the pH in the ceca [14]. The cecal microbiota ferments undigested carbohydrates, producing VFAs, and modulates immunity [15]. Moreover, a balanced cecal microbiota may improve gut integrity, pathogen resistance, and overall bird health [16]. Recent research has shown that insect-derived fats, such as BSFL fat, which are used as partial or total substitutes for soybean oil (SO), have no negative effects on the cecal microbiome in broilers [17]. However, the effects of BSFL fat on the GIT environment and cecal microbiome relative to those of various animal fats and plant oils remain underexplored. This study is based on the hypothesis that BSFL fat positively affects the GIT pH and cecal microbiome and has effects comparable to those of plant oils because of the high level of C12:0 present. Therefore, this study aims to compare the effects of BSFL fat with those of selected dietary fats of plant and animal origin in broiler diets on the GIT environment and cecal microbiome structure.

## Materials and methods

### Ethics statement

These experimental procedures used herein did not require approval from the Local Ethical Committee for Experiments on Animals in Poznań, in compliance with Polish law (Journal of Law, 2015, item 266) and EU Directive (2010/63/EU) [18] on the protection of animals used for scientific purposes. All procedures were conducted in accordance with the guidelines of the Local Ethics Commission of Poznań University of Life Sciences (Poznań, Poland) for animal experimentation and care. No action involving pain or suffering was performed, and all analyses were performed on postmortem samples. Sacrificing animals solely for the use of their organs and tissues is considered an acceptable practice. The directive states the requirements for protecting animals used for experimental purposes. Therefore, these rules do not apply to agricultural activities or to animal husbandry. The experiment was conducted under commercial conditions, and the farmers were responsible for rearing the animals.

### Housing

A total of 800 one-day-old male Ross 308 chicks (initial weight 40 ± 0.5 g) were obtained from a commercial hatchery. The chicks were randomly assigned to eight dietary groups, with each group consisting of 10 replicate pens containing 10 birds each, and the study lasted for 35 days. The broilers were housed in 1 m × 1 m floor pens with chopped wheat straw (7–15 cm) used as bedding material. The experiment was conducted in chicken house number 0161 in Olszowa, Poland, with pens surrounded by 9,000 unsexed broilers of the same origin to replicate intensive farming conditions. The facility was equipped with programmable fluorescent lighting, automatic electric heating, and forced ventilation, in line with the Aviagen: Ross Broiler Management Handbook [19] and Council Directive (2007/43/EC) [20].

### Experimental diets

The composition and nutritional value of the eight experimental diets used in this study, which were divided into two phases, starter (up to 14 days) and grower (15–35 days), are provided in Tables 1 and 2. The aim in this study was to enrich basal diets by incorporating selected fat sources, including BSFL fat, soybean oil (SO), rapeseed oil (RO), palm oil (PO), palm kernel fatty acid distillate (PKFD), poultry fat (PF), pig lard (PL), and beef tallow (BT). The diets were formulated by Piast Pasze Sp. z o.o. (Lewkowiec, Poland) following ISO 9001:2008 standards and optimized to meet or surpass the nutrient requirements of broilers, as recommended by Aviagen [21]. Because each dietary fat was included at the same level in the experimental diets and other ingredients were not modified, the diets were not isoenergetic and were provided throughout the 1–35 day rearing period. The apparent metabolizable energy (AME) of BSFL fat for broilers was determined using the regression model by Kierończyk et al. 2022 [6], while the AME values of the other dietary fats were sourced from INRAE-CIRAD-AFZ (2021) [22]. Insect production and fat extraction were performed following the methods described by Kierończyk et al. [23]. The experimental diets were prepared without heat treatment and contained no additives, such as exogenous enzymes, emulsifiers, or coccidiostats. During the entire experimental period, the broiler chickens had ad libitum access to both feed and water.

**Table 1:** Composition and nutritive value of starter diets (1–14 days).

Ingredients, %	^1^BSFL	^2^SO	^3^RO	^4^PO	^5^PKFD	^6^PF	^7^PL	^8^BT
Maize	55.0	55.0	55.0	55.0	55.0	55.0	55.0	55.0
Soybean meal	37.3	37.3	37.3	37.3	37.3	37.3	37.3	37.3
Dietary fat ^a^	3.5	3.5	3.5	3.5	3.5	3.5	3.5	3.5
Vitamin premix *	0.3	0.3	0.3	0.3	0.3	0.3	0.3	0.3
Dicalcium phosphate	2.6	2.6	2.6	2.6	2.6	2.6	2.6	2.6
Limestone	0.35	0.35	0.35	0.35	0.35	0.35	0.35	0.35
NaCl	0.27	0.27	0.27	0.27	0.27	0.27	0.27	0.27
Na_2_SO_4_	0.13	0.13	0.13	0.13	0.13	0.13	0.13	0.13
L-lysine	0.12	0.12	0.12	0.12	0.12	0.12	0.12	0.12
L-methionine	0.27	0.27	0.27	0.27	0.27	0.27	0.27	0.27
L-threonine	0.09	0.09	0.09	0.09	0.09	0.09	0.09	0.09
L-valine	0.03	0.03	0.03	0.03	0.03	0.03	0.03	0.03
Calculated nutritive value, %								
Crude protein	21.9	21.9	21.9	21.9	21.9	21.9	21.9	21.9
Crude fat	6.1	6.1	6.1	6.1	6.1	6.1	6.1	6.1
Crude fiber	2.7	2.7	2.7	2.7	2.7	2.7	2.7	2.7
Calcium	0.9	0.9	0.9	0.9	0.9	0.9	0.9	0.9
Lysine	1.3	1.3	1.3	1.3	1.3	1.3	1.3	1.3
Methionine+cystine	0.98	0.98	0.98	0.98	0.98	0.98	0.98	0.98
^9^AME, MJ/kg	12.6	12.6	12.6	12.3	12.4	12.5	12.4	12.3

^a^The following dietary fats were added: ^1^BSFL = *Hermetia illucens* larvae fat; ^2^SO = soybean oil; ^3^RO = rapeseed oil; ^4^PO = palm oil; ^5^PKFD = palm kernel fat distillate; ^6^PF = poultry fat; ^7^PL = pork lard; ^8^BT = beef tallow; ^9^AME = apparent metabolizable energy *Provided the following per kilogram of diet: vitamin A, 11 000 IU; cholecalciferol, 2 500 IU; vitamin E, 50 mg; menadione, 2.50 mg; vitamin B, 0.02 mg; folic acid, 1.0 mg; choline, 300 mg; D- pantothenic acid, 13.6 mg; riboﬂavin, 7.0 mg; niacin, 41.7 mg; thiamine, 2.0 mg; D-biotin, 0.20 mg; pyridoxine, 4.0 mg; ethoxyquin, 0.1 mg; Mn (MnO_2_), 60 mg; Zn (ZnO), 95 mg; Fe (FeSO_4_), 45 mg; Cu (CuSO_4_), 20 mg; I (CaI_2_O_6_), 0.6 mg; and Se (Na_2_SeO_3_), 0.35 mg.

**Table 2:** Composition and nutritive value of grower diets (15–35 days).

Ingredients, %	^1^BSFL	^2^SO	^3^RO	^4^PO	^5^PKFD	^6^PF	^7^PL	^8^BT
Maize	63.5	63.5	63.5	63.5	63.5	63.5	63.5	63.5
Soybean meal	28.4	28.4	28.4	28.4	28.4	28.4	28.4	28.4
Dietary fat ^a^	5.0	5.0	5.0	5.0	5.0	5.0	5.0	5.0
Vitamin premix*	0.3	0.3	0.3	0.3	0.3	0.3	0.3	0.3
Dicalcium phosphate	1.2	1.2	1.2	1.2	1.2	1.2	1.2	1.2
Limestone	0.4	0.4	0.4	0.4	0.4	0.4	0.4	0.4
NaCl	0.3	0.3	0.3	0.3	0.3	0.3	0.3	0.3
Na_2_SO_4_	0.1	0.1	0.1	0.1	0.1	0.1	0.1	0.1
L-lysine	0.2	0.2	0.2	0.2	0.2	0.2	0.2	0.2
L-methionine	0.3	0.3	0.3	0.3	0.3	0.3	0.3	0.3
L-threonine	0.2	0.2	0.2	0.2	0.2	0.2	0.2	0.2
L-valine	0.1	0.1	0.1	0.1	0.1	0.1	0.1	0.15
Calculated nutritive value, %								
Crude protein	18.6	18.6	18.6	18.6	18.6	18.6	18.6	18.6
Crude fat	7.7	7.7	7.7	7.7	7.7	7.7	7.7	7.7
Crude fiber	2.5	2.5	2.5	2.5	2.5	2.5	2.5	2.5
Calcium	0.7	0.7	0.7	0.7	0.7	0.7	0.7	0.7
Lysine	0.9	0.9	0.9	0.9	0.9	0.9	0.9	0.9
Methionine+cystine	0.9	0.9	0.9	0.9	0.9	0.9	0.9	0.9
^9^AME, MJ/kg	13.4	13.4	13.4	13.0	13.2	13.4	13.2	13.0

^a^The following dietary fats were added: ^1^BSFL = *Hermetia illucens* larvae fat; ^2^SO = soybean oil; ^3^RO = rapeseed oil; ^4^PO = palm oil; ^5^PKFD = palm kernel fat distillate; ^6^PF = poultry fat; ^7^PL = pork lard; ^8^BT = beef tallow; ^9^AME = apparent metabolizable energy *Provided the following per kilogram of diet: vitamin A, 11 000 IU; cholecalciferol, 2 500 IU; vitamin E, 50 mg; menadione, 2.50 mg; vitamin B, 0.02 mg; folic acid, 1.0 mg; choline, 300 mg; D- pantothenic acid, 13.6 mg; riboﬂavin, 7.0 mg; niacin, 41.7 mg; thiamine, 2.0 mg; D-biotin, 0.20 mg; pyridoxine, 4.0 mg; ethoxyquin, 0.1 mg; Mn (MnO_2_), 60 mg; Zn (ZnO), 95 mg; Fe (FeSO_4_), 45 mg; Cu (CuSO_4_), 20 mg; I (CaI_2_O_6_), 0.6 mg; and Se (Na_2_SeO_3_), 0.35 mg.

### Data and sample collection

A total of 80 birds (one randomly selected from each replicate pen, n = 10) were subjected to electrical stunning (STZ-6, PPHU KOMA, Poland) at the end of the experiment (day 35), followed by sacrifice (cervical dislocation) and evisceration. Stunning prior to sacrifice ensured loss of consciousness and minimized any potential suffering. Digesta samples were collected from the crop, gizzard, jejunum (beginning at the end of the duodenum and ending at Meckel’s diverticulum), and ceca. pH was measured using a 1100 H pH meter (VWR International, Leuven, Belgium) with a ScienceLine Micro pH combination electrode N 6000 BNC (Schott SI Analytics, Mainz, Germany). Triplicate measurements were taken from each GIT segment of each bird, and the mean value was used for further calculations (*n* = 10). Cecal digesta samples were collected directly in Eppendorf tubes and immediately frozen for microbial analysis.

### DNA extraction, amplification, and sequencing

Following the manufacturer’s instructions, DNA was extracted from the cecal digesta samples (300 ± 10 mg per sample) via the Genomic Mini AX Bacteria + (A&A Biotechnology, Gdynia, Poland). Digesta samples were pooled from two individual birds (*n* = 5). First, the samples were mechanically lysed via a FastPrep-24 instrument with Lysing Matrix A (MP Biomedicals, Santa Ana, CA, USA). After isolation, the DNA was further purified using an anti-inhibitor kit (A&A Biotechnology, Gdynia, Poland). The presence of bacterial DNA was detected via real-time PCR on an Mx3000P thermocycler (Stratagene, USA) with SYBR Green as the fluorochrome. The universal primers 1055F (5’-ATGGCTGTCGTCAGCT-3’) and 1392R (5’-ACGGGCGGTGTGTAC-3’) were used to amplify 16S rDNA with the following temperature program: 3 min at 95°C; 15 sec at 95°C, 30 sec at 58°C, and 30 sec at 72°C; and a melting curve step from 65°C to 95°C. Finally, bacterial DNA was quantified using a NanoDrop™ One Microvolume UV–Vis spectrophotometer (Thermo Fisher Scientific, Waltham, MA, USA) and standardized to 5 ng/μL. The sequencing analysis was performed by GENOMED S.A. (Warsaw, Poland). In brief, microbiome diversity was assessed by sequencing the amplified V3-V4 region of the 16S rRNA gene via the primers 16S Amplicon PCR Forward Primer (5’- TCGTCGGCAGCGTCAGAT-GTGTATAAGAGACAGCCTACGGGNGGCWGCAG) and 16S Amplicon PCR Reverse Primer (5’-GTCTCGTGGGCTCGGAGATGTGTATAAGAGACAGGACTACHVGGGTATC-TAATCC). The amplification conditions were as follows: 3 min at 95°C; 25 cycles of 30 s at 95°C, 30 s at 55°C, and 30 s at 72°C; 5 min at 72°C; hold at 4°C. The expected amplicon size was approximately 550 bp; the PCR products were cleaned with AMPure XP beads. The libraries were sequenced using 2 × 300 bp paired-end reads, and the cleaned PCR products were combined with sequencing adapters and dual indicators using the Nextera XT Index Kit (Illumina, San Diego, USA) following the 16S Metagenomic Sequencing Library Preparation instructions. The conditions for the PCR assay with the Nextera XT Index Primer were as follows: 3 min at 95°C; eight cycles of 30 s at 95°C, 30 s at 55°C, and 30 s at 72°C; 5 min at 72°C; and hold at 4°C. The PCR products were then purified using AMPure XP beads, resulting in a final library size of approximately 630 bp, as validated using a Bioanalyzer. The libraries were quantified using a fluorometric method that utilizes dsDNA-binding dyes, and the individual concentrations of the DNA libraries were determined in nM on the basis of the size of the DNA amplicons assessed using an Agilent 2100 Bioanalyzer (Agilent Technologies, Santa Clara, CA, USA). For sequencing, the individual libraries were adjusted to a concentration of 4 nM, denatured with 10 mM Tris (pH 8.5), and supplemented with 20% (v/v) PhiX. A 5 μL aliquot of the diluted DNA was subsequently mixed to pool the library preparations for MiSeq (Illumina, San Diego, USA) runs, yielding more than 100,000 sample reads. Bioinformatic analysis was performed as described by Trela et al. [24]. Sequencing reads were normalized using Total Sum Scaling (TSS) to relative abundance for all downstream analyses of community composition and relative abundance. This normalization approach was selected as it is standard for compositional microbiome data, and preliminary analysis confirmed that library sizes were consistent across experimental groups, minimizing potential artifacts related to sequencing depth [25].

### Statistical analysis

The experimental design employed complete randomization, designating the replicate pen (*n* = 10) as the experimental unit for measuring the pH of the GIT digesta. However, for next-generation sequencing analysis, pooled cecal digesta from two randomly chosen birds were used as the experimental unit (*n* = 5). RStudio (2024.04.2 + 764; Posit, PBC, Boston, MA, USA) was used for the statistical analyses. The normality and homogeneity of variances of the data were assessed using the Shapiro‒Wilk test and Levene’s test, respectively. For normally distributed data, one-way ANOVA and Dunnett’s post hoc test were used to compare multiple treatments to the reference group (BSFL), whereas nonnormally distributed data were analyzed using the Kruskal‒Wallis test followed by Dunn’s test with Benjamini‒Hochberg adjustment, with significant differences considered at P < 0.05.

Further analyses of *α* diversity (diversity within samples) and *β* diversity (similarity between different microbiome profiles) were performed using the *vegan* package (v2.6-8). Four *α* diversity indices were calculated, namely, the Shannon, Simpson, richness, and evenness indices. *β-*Diversity was assessed using the Bray‒Curtis dissimilarity matrix and evaluated for the multivariate effects of different fat types on species-level bacterial communities via nonparametric permutational multivariate analysis of variance (PERMANOVA) with *adonis2* with 999 permutations (for significant differences in community composition across groups) and pairwise *adonis* (pairwise comparisons between specific groups) functions, whereas bacterial profiles at the species level among the different groups were visualized using nonmetric multidimensional scaling (NMDS). The differential abundance of the cecal microbiota was determined via linear discriminant analysis effect size (LEfSe) using the *microbiomeMarker* (v1.10.0) package. Using presample normalization of the summed values to 1e + 06, we applied the Kruskal‒Wallis and Wilcoxon tests with a significance level of P < 0.05, along with a linear discriminant analysis (LDA) threshold value of 2. The results were then visually represented on the basis of the Log10 (LDA score).

The differences between treatments were calculated using two approaches: separately for each dietary treatment versus BSFL and collectively for plant oils and animal fats versus BSFL.

## Results

### Digesta pH

Table 3 presents the pH values of the digesta of the selected GIT segments. PKFD, PF, and BT significantly increased the pH of the crop (P = 0.005) compared to BSFL, whereas PKFD reduced (P = 0.006) the pH of the gizzard relative to BSFL. No statistically significant differences were detected among the effects of the experimental diets compared with that of BSFL on the pH in the jejunum (P = 0.012 ANOVA, P > 0.05, post hoc test) or ceca (P = 0.053).

**Table 3:** Effects of the inclusion of selected types of dietary fat in broiler chicken diets on the gastrointestinal tract pH.

	Treatments								^9^SEM	ANOVA P value
	^1^BSFL	^2^SO	^3^RO	^4^PO	^5^PKFD	^6^PF	^7^PL	^8^BT		
Crop	4.31	4.77	4.54	4.97	5.16*	5.42**	4.99	5.12*	0.126	0.005
Gizzard	3.35	2.86	2.86	3.24	2.48*	2.80	2.95	2.95	0.095	0.006
Jejunum	5.82	5.72	5.79	5.45	5.99	5.82	5.61	5.57	0.060	0.012
Ceca	6.64	6.68	6.66	6.89	6.11	6.45	6.69	6.58	0.079	0.053

* p < 0.05, ** p < 0.01, *** p < 0.001: asterisks indicate significance differences from the reference group, i.e., BSFL. ^1^BSFL – basal diet with 100% black soldier fly (*Hermetia illucens*) larval fat; ^2^SO – basal diet with 100% soybean oil; ^3^RO – basal diet with 100% rapeseed oil; ^4^PO – basal diet with 100% palm oil; ^5^PKFD – basal diet with 100% palm kernel fat distillate; ^6^PF – basal diet with 100% poultry fat; ^7^PL – basal diet with 100% pig lard; ^8^BT – basal diet with 100% beef tallow; ^9^SEM – standard error of the mean.

### Diversity and taxonomical abundance

NGS analyses were performed on 40 samples to generate 4,502,160 raw sequence reads. After quality filtering, 3,777,005 (83,9%) sequences were obtained. The relative abundance of bacteria was recorded in all experimental groups (100%).

**Fig 1:**
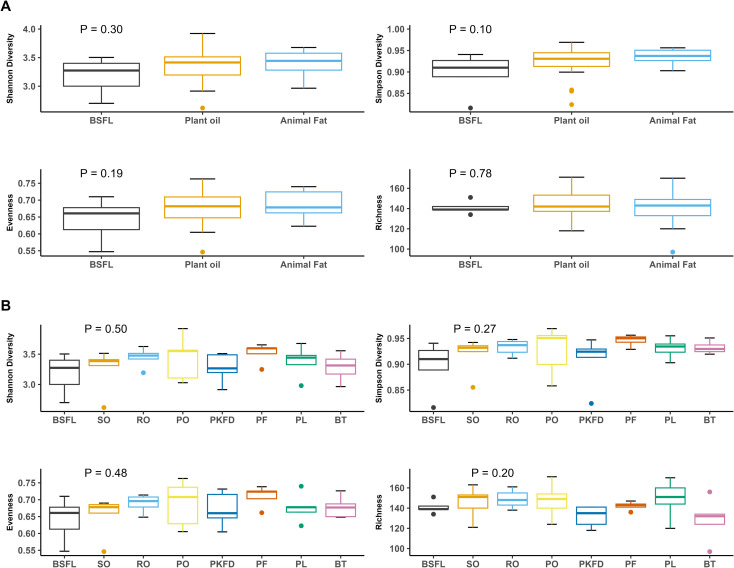
Diversity analysis of microbial communities in broiler ceca. (A) metrics (Shannon diversity, Simpson diversity, evenness, and richness) across three groups (BSFL, plant oils, and animal fats); (B) alpha diversity metrics for eight diets featuring various fat sources. BSFL – basal diet with 100% black soldier fly (*Hermetia illucens*) larval fat; plant oil diets (SO – basal diet with 100% soybean oil; RO – basal diet with 100% rapeseed oil; PO – basal diet with 100% palm oil; PKFD – basal diet with 100% palm kernel fat distillate); animal fats (PF – basal diet with 100% poultry fat; PL – basal diet with 100% pig lard; BT – basal diet with 100% beef tallow).

**Fig 2:**
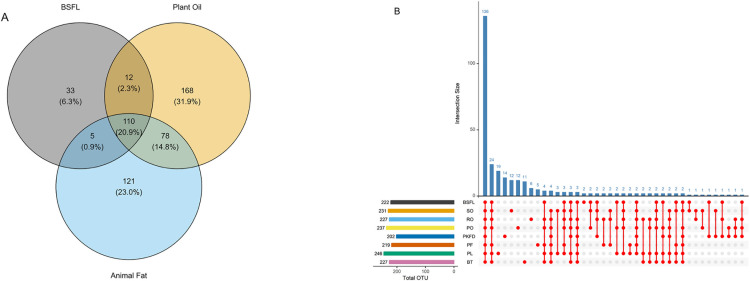
(A) Venn diagram of the overlap among three groups (BSFL, plant oils, and animal fats); (B) UpSet plot representing the intersection of OTUs across eight experimental groups in the ceca of broiler chickens. BSFL – basal diet with 100% black soldier fly (*Hermetia illucens*) larval fat; plant oils (SO – basal diet with 100% soybean oil; RO – basal diet with 100% rapeseed oil; PO – basal diet with 100% palm oil; PKFD – basal diet with 100% palm kernel fat distillate); animal fats (PF – basal diet with 100% poultry fat; PL – basal diet with 100% pig lard; BT – basal diet with 100% beef tallow).

**Fig 3:**
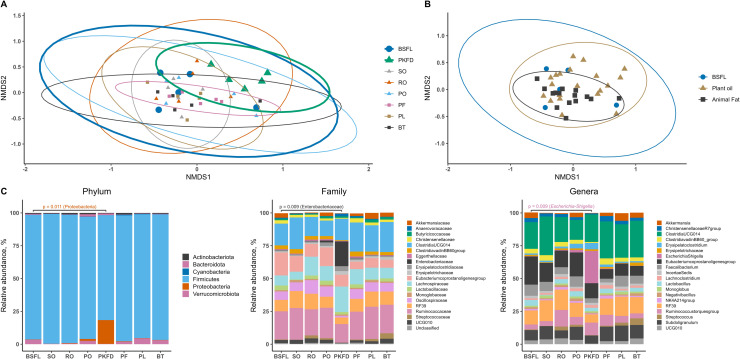
Graphical representation of beta diversity analysis and bacterial abundance in broiler ceca. (A) nonmetric multidimensional scaling (NMDS) plot of beta diversity among individual fat types in the cecal microbiota of broilers at the species level; (B) NMDS plot of beta diversity among three groups (BSFL, plant oils, and animal fats) in the cecal microbiota of broilers at the species level; (C) distribution of cecal bacteria at different taxonomic levels (phylum, family, and genus) among various fat types, including the top 20 families and genera. BSFL – basal diet with 100% black soldier fly (*Hermetia illucens*) larval fat; plant oils (SO – basal diet with 100% soybean oil; RO – basal diet with 100% rapeseed oil; PO – basal diet with 100% palm oil; PKFD – basal diet with 100% palm kernel fat distillate); animal fats (PF – basal diet with 100% poultry fat; PL – basal diet with 100% pig lard; BT – basal diet with 100% beef tallow).

Fig 1 shows the *α* diversity of the microbiota in the cecal digesta, comparing the three groups collectively and individually, with comparisons made relative to BSFL. No significant differences (P > 0.05) were detected between the reference group and the other dietary treatment groups in terms of *α* diversity. The Venn diagram (Fig 2A) illustrates the overlap in microbial taxa among the BSFL, plant oil, and animal fat groups. A total of 20.9% of the taxa were shared across all three fat types, 2.3% were shared exclusively between BSFL and plant oil, and 0.9% were shared between BSFL and animal fat. In addition, 6.3% of the taxa were unique to the BSFL group. Fig 2B shows the intersection of the OTUs across the eight groups. Among the 222 OTUs present in the BSFL group, 136 OTUs were shared across all the experimental groups, indicating that these taxa were common to all the treatments. Additionally, 24 OTUs were shared across all groups except PKFD, and 2 OTUs were exclusive to BSFL. Additionally, for the three groups (BSFL, plant oils, and animal fats), no significant differences were observed in the *β* diversity (P = 0.36). In contrast, for the eight groups, the Bray‒Curtis dissimilarity was significant (P = 0.046), with the only notable difference observed between BSFL and PKFD (P = 0.034). Nonmetric multidimensional scaling (NMDS) of the Bray‒Curtis dissimilarity matrix is shown in Fig 3(A and B).

Fig 3C and S1 Table shows the abundance of microbial taxa at the phylum level and the top 20 taxa at the family and genus levels. Analysis of the OTU table revealed that the abundance of the phylum Proteobacteria was greater (P = 0.011) in PKFD than in BSFL. A similar result was observed at the family and genus levels, where compared with BSFL, PKFD increased the abundance of Enterobacteriaceae (P = 0.009) and *Escherichia/Shigella* (P = 0.009).

### LEfSe analysis

**Fig 4:**
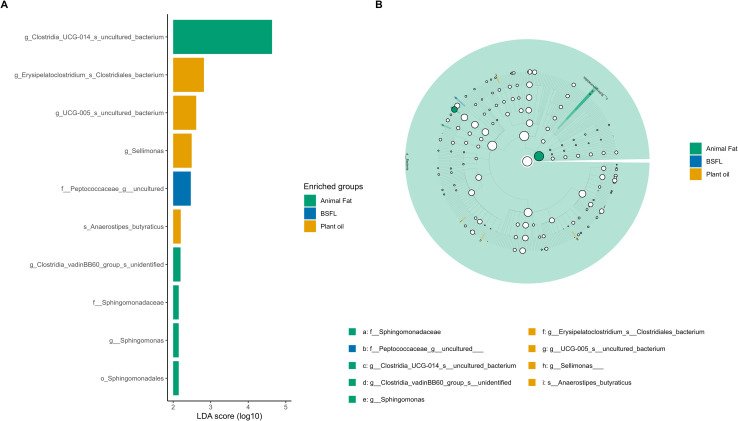
Linear discriminant analysis effect size (LEfSe) at various taxonomic levels (kingdom (k), phylum (p), class (c), order (o), family (f), genus (g), and species (s)) for broiler chickens fed diets containing fats of plant, animal, and insect origin. (A) bar graph of LDA scores for three groups (BSFL, plant oil, and animal fats), representing log10-transformed values for differentially abundant taxa; (B) cladogram illustrating phylogenetic relationships between taxa with significant differences in abundance across three groups (BSFL, plant oil, and animal fats). BSFL – basal diet with 100% black soldier fly (*Hermetia illucens*) larval fat; plant oils (SO – basal diet with 100% soybean oil; RO – basal diet with 100% rapeseed oil; PO – basal diet with 100% palm oil; PKFD – basal diet with 100% palm kernel fat distillate); animal fats (PF – basal diet with 100% poultry fat; PL – basal diet with 100% pig lard; BT – basal diet with 100% beef tallow).

**Fig 5:**
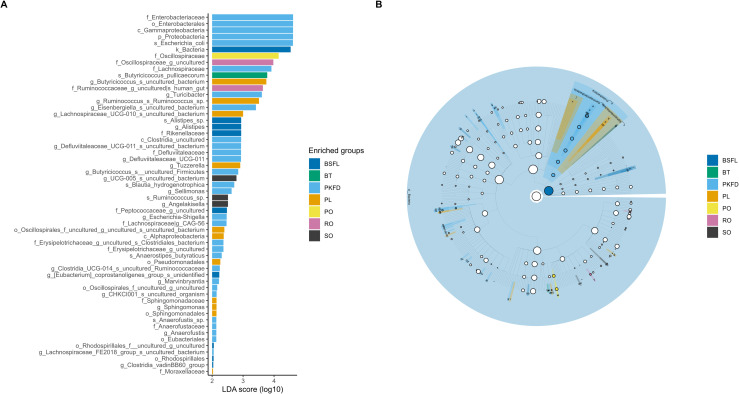
Linear discriminant analysis effect size (LEfSe) at various taxonomic levels (kingdom (k), phylum (p), class (c), order (o), family (f), genus (g), and species (s)) for broiler chickens fed diets containing fats of plant, animal, and insect origin. (A) bar graph of LDA scores for individual fats, representing log10-transformed values for differentially abundant taxa; (B) cladogram illustrating phylogenetic relationships between taxa with significant differences in abundance across different diets. BSFL – basal diet with 100% black soldier fly (*Hermetia illucens*) larval fat; SO – basal diet with 100% soybean oil; RO – basal diet with 100% rapeseed oil; PO – basal diet with 100% palm oil; PKFD – basal diet with 100% palm kernel fat distillate; PL – basal diet with 100% pig lard; BT – basal diet with 100% beef tallow.

Fig 4 and S2 Table presents the LDA results, highlighting the effect size of each marker and the phylogenetic relationships between taxa with significant differences in abundance across the three groups. BSFL had only a single microbial marker, that is, Peptococcaceae [uncultured] (P = 0.019). Fig 5 and S3 Table shows the results of the LDA, which revealed significant differences in abundance across the eight groups. The most significant taxa in the BSFL group were total bacteria (P < 0.001), Rikenellaceae (P = 0.025), Peptococcaceae [uncultured genus] (P = 0.003), Rhodospirillales (P = 0.048), *Alistipes* (P = 0.025), *Eubacterium coprostanoligenes* (P = 0.018), *Clostridia vadin BB*60 (P = 0.032), uncultured *Rhodospirillales* (P = 0.048), and *Alistipes* sp. (P = 0.023).

## Discussion

This study compared the effects of including BSFL fat in broiler chicken diets with those of commonly used plant- and animal-derived fats on the GIT environment and cecal microbiome. The balance between saturated and unsaturated fats can influence digesta viscosity, microbial composition, and pH, thereby affecting digestive efficiency and pathogen inhibition [3]. Given the unique medium-chain fatty acid profile of BSFL fat, its impact on the GIT pH compared with that of commonly used dietary fats warrants further investigation. Moreover, although previous studies have examined BSFL fat in comparison with soybean oil [26], its effects on the cecal microbiota compared with a broader range of fat sources remain unexplored. Therefore, this study evaluated the influence of BSFL fat relative to other dietary fats on pH dynamics across different GIT segments and the cecal microbiota. Due to the previous research compared the effect of BSFL fat with common dietary fat sources in broiler chicken diets on cecal bacteria using fluorescence in situ hybridization [9], the present study employs next-generation sequencing for a more comprehensive assessment of BSFL fat relative to multiple dietary fat sources on cecal microbiota and pH dynamics across the GIT. The pH of the crop in the BSFL group was lower than that in the PKFD, PF, and BT groups, likely due to prolonged retention time and antiperistaltic movement. Low crop pH is known to inhibit various pathogenic and zoonotic microorganisms, such as *Salmonella*, *Campylobacter*, and *Clostridia* [27]. This acidic environment also promotes organic acid production by the crop microbiota, further reducing pH and reinforcing microbial inhibition [28]. Among these organic acids, butyric acid is more effective at low pH because its undissociated form can cross bacterial membranes, acidify the cytoplasm, and exert strong antimicrobial effects [29]. While BSFL fat does not differ from most plant oils, such as SO, RO, or PO, previous research suggests that BSFL fat reduces crop pH when it fully replaces SO in broiler diets [8]. However, more recent findings by Kierończyk et al. [30] indicate that this replacement may have no effect on crop pH. Interestingly, BSFL increased the pH in the gizzard compared with PKFD, whereas no difference relative to BSFL was observed in the other groups. Although the pH for both groups remained within the physiological range (2.5–3.5), this should not be considered detrimental. Limited data exist on the influence of BSFL, compared with other fat sources, on gizzard pH. Nevertheless, studies suggest that complete or partial replacement of SO with BSFL fat reduces gizzard mass [30]. However, no significant differences in gizzard weight with similar replacements were reported in another study [31]. These varying results highlight the need for further investigation to clearly explain this phenomenon.

Although C12:0 is well known for its antimicrobial properties [32], this study revealed that compared with other fat sources, BSFL fat had minimal effects on the *α*- and *β*-diversity or taxonomic abundance of bacteria in the cecal microbiome, except for differences between BSFL and PKFD in terms of *β*-diversity and the abundance of Proteobacteria, Enterobacteriaceae, and *Escherichia–Shigella*. These populations are known to contribute to foodborne illness and antimicrobial resistance, which are major public health concerns [33]. This finding is in line with those of Schäfer et al. [17], who reported no effect on *α*- and *β*-diversity or on the abundance of taxa when SO was completely substituted with BSFL. The reduction in the bacterial community (Proteobacteria, Enterobacteriaceae, and *Escherichia-Shigella*) abundance observed with BSFL compared with PKFD was likely due to variations in the pH of the crop and gizzard rather than differences in FA composition. Notably, PKFD had an FA composition similar to that of SO and RO [9]; however, our study revealed no significant effects of BSFL compared with either SO or RO. The above hypothesis was supported by the findings of Projahn et al. [34], who reported that a relatively high crop pH could result in an increase in Enterobacteriaceae abundance. Further research comparing BSFL fat with plant and animal fats across the GIT could clarify how pH and fat type affect the gut microbiome.

In particular, the bacterial community in the ceca was dominated by Firmicutes across all dietary fat groups, which is consistent with the findings of Chen et al. [35], who replaced SO with insect oil. At the family level, the dominant taxa were Ruminococcaceae, Clostridia UCG-014, Lachnospiraceae, and *Eubacterium coprostanoligenes* (>50%). Ruminococcaceae family is known primarily for its ability to produce short-chain fatty acids (SCFAs), especially butyrate, which is essential for gut health and possesses anti-inflammatory properties, whereas Lachnospiraceae also produces SCFAs by converting lactate to butyrate, whereas *Clostridia* UCG014 promotes growth in broiler chickens by competing with pathogenic bacteria such as *Clostridium perfringens*, which is responsible for causing necrotic enteritis [36]. The *Eubacterium coprostanoligenes* group participates in cholesterol metabolism by converting cholesterol to coprostanol and producing SCFAs, such as acetic, formic, and succinic acids [37]. In general, coprostanol is less absorbable by the gut than cholesterol is, which could reduce cholesterol reabsorption and potentially improve overall health. The top genera across all experimental groups were *Subdoligranulum*, *RF*39, and unclassified genera in the *Clostridia* UCG-014 and *Eubacterium coprostanoligenes* families, which is in contrast to the findings of Dabbou et al. [38], who reported increases in the abundances of *Clostridium*, *Lactobacillus*, and Peptostreptococcaceae associated with BSFL fat compared with SO. Moreover, RF39 is known to be involved in the production of acetate and hydrogen, which are important for gut health [39], whereas *Subdoligranulum* participates in fiber degradation and SCFA production, particularly butyrate production [40]. The lack of differences between the BSFL and other experimental diets, except PKFD at the family and genus levels, was surprising and contradicts the findings of Deryabin et al. [41], who reported that, compared with UFAs, SFAs have an adverse effect on cecal bacteria. It was assumed that the C12:0 present in BSFL did not reach a minimum inhibitory concentration in the ceca because of the efficient absorption of MCFAs in the small intestine. This assumption is supported by a previous study in which the addition of MCFA (C6:0, C8:0, C10:0) did not decrease *Campylobacter* colonization in broiler ceca [42]. However, Szabó et al. [10] reported that the inclusion of MCFAs in broiler diets can reduce the population of coliforms and other enteric pathogens in the ceca. Although C12:0 had no significant effect on the bacterial population abundance in the ceca, its influence on the upper parts of the gastrointestinal tract (GIT) may play a role in shaping the cecal microbiome.

LEfSe analysis highlighted the differential abundance of distinct taxa among all experimental treatments, except for PF. BSFL increased the total bacterial population compared with the other treatments, particularly the abundances of Rhodospirillales, Peptococcaceae, the *Clostridia vadin BB*60 group, the *Eubacterium coprostanoligenes* group and *Alistipes* sp. This result contrasts with the findings of Chen et al. [35], who reported that the complete replacement of SO with BSFL fat increased the abundances of the families Veillonellaceae and Staphylococcaceae and of *Hegamonas*. Rhodospirillales belongs to Proteobacteria, members of which play a key role in fermentation processes, particularly in SCFA production and nitrogen recycling from uric acid, and support protein synthesis and the overall nitrogen balance in birds [41,43]. Peptococcaceae species produce propionate and support gut health by competitively excluding pathogens, showing probiotic potential that may reduce antibiotic use in poultry [44]. The *Clostridiales vadin* BB60 group is capable of degrading complex plant polysaccharides and contributes to intramuscular fat deposition in broiler chickens without affecting growth performance or meat yield [45]. *Alistipes* species play important roles in the production of SCFAs, such as butyrate, acetate, and propionate [46]. In addition to their metabolic functions, *Alistipes* species may aid in the maturation of the immune system, enhancing the body’s ability to combat pathogens while promoting a balanced inflammatory response. The anti-inflammatory properties of these compounds may further mitigate inflammation through the production of sulfonolipids [11]. The presence of different markers in BSFL compared with conventionally used fat sources suggests that different FA profiles could have specific impacts on the composition of the cecal microbiome, highlighting the complexity of the dependencies between dietary fats and microbial communities. While these findings provide valuable insights into the effects of dietary fat on the GIT and cecal microbiota, it is important to consider the limitations of the experimental design. The main aim of this study was to determine the effect of dietary fat type by formulating diets that were identical except for their fatty acid profile. This design allowed the observed responses of the cecal microbiota to be linked more directly to the fat source. A limitation, however, was that the diets were not strictly isocaloric, since the AME of the different fats was not adjusted. As a result, the outcomes may reflect both differences in fat type and variation in dietary energy. Future studies using isoenergetic diets, with the AME of each fat source carefully standardized, are needed to clarify the specific role of fat type on performance and gut micro-environment.

## Conclusion

The comparison of BSFL fat application in broiler chicken diets with a wide spectrum of commonly used dietary fats revealed differences between insect and selected animal fats in terms of crop environment modulation. Furthermore, no changes were observed in the cecal microbiome diversity or the abundance of most taxa within the BSFL treatment group compared with the other experimental groups. Nevertheless, BSFL showed the most significant and beneficial differences in reducing the proliferation of Proteobacteria, Enterobacteriaceae, and *Escherichia/Shigella*, in contrast to PKFD. Additionally, the differential abundance of distinct bacterial populations was observed when *H. illucens* fat was used as an energy source, promoting the growth of the total bacterial population and increasing the abundances of Rikenellaceae, Peptococcaceae, Rhodospirillales, *Alistipes*, the *Eubacterium coprostanoligenes* group, and the *Clostridia vadin* BB60 group, which are associated with enhanced SCFA production, improved gut health, nutrient utilization, and immune modulation in broilers. Compared with that of other dietary fats, the application of insect fat in broiler chicken diets does not have a strong effect on the GIT environment or cecal microbiota. However, it may support beneficial microbial communities that are associated with nutrient utilization and gut health.

## Supporting information

S1 Table Relative abundance of microbiota at the phylum level, and the top 20 dominant families and genera in cecal digesta samples. (DOCX)

S2 Table Biomarker identification in broiler chicken ceca via LEfSe analysis (3 groups). (DOCX)

S3 Table Biomarker identification in broiler chicken ceca via LEfSe analysis (8 groups). (DOCX)
