# Supplementary material for: Cecal microbiota of broilers responds similarly to black soldier fly larvae fat and conventional dietary fat sources
Source: PLoS One. 2025 Nov 25;20(11):e0336523. doi: 10.1371/journal.pone.0336523 (PMC12646438; doi:10.1371/journal.pone.0336523)
Supplement: S1 Table — (DOCX) [file pone.0336523.s001.docx]

**S1 Table.** Relative abundance of microbiota at the phylum level, and the top 20 dominant families and genera in cecal digesta samples.

|  | Treatment | | | | | | | | ^9^SEM | P value |
| --- | --- | --- | --- | --- | --- | --- | --- | --- | --- | --- |
|  | ^1^BSFL | ^2^SO | ^3^RO | ^4^PO | ^5^PKFD | ^6^PF | ^7^PL | ^8^BT |  |  |
| **Phyla** | | | | | | | | | | |
| Actinobacteriota | 0.006 | 0.006 | 0.010 | 0.010 | 0.004 | 0.011 | 0.005 | 0.006 | 0.001 | 0.427 |
| Bacteroidota | 0.008 | 0.002 | 0.003 | 0.018 | 0.013 | 0.002 | 0.002 | 0.004 | 0.002 | 0.277 |
| Cyanobacteria | 0.001 | 0.001 | 0.002 | 0.002 | 0.000 | 0.005 | 0.003 | 0.001 | 0.001 | 0.220 |
| Firmicutes | 0.949 | 0.988 | 0.976 | 0.930 | 0.799 | 0.958 | 0.943 | 0.956 | 0.021 | 0.107 |
| Proteobacteria | 0.002 | 0.001 | 0.004 | 0.014 | 0.181** | 0.004 | 0.002 | 0.002 | 0.022 | 0.011 |
| Verrucomicrobiota | 0.034 | 0.003 | 0.005 | 0.025 | 0.003 | 0.019 | 0.045 | 0.031 | 0.006 | 0.861 |
| Actinobacteriota | 0.006 | 0.006 | 0.010 | 0.010 | 0.004 | 0.011 | 0.005 | 0.006 | 0.001 | 0.427 |
| Bacteroidota | 0.008 | 0.002 | 0.003 | 0.018 | 0.013 | 0.002 | 0.002 | 0.004 | 0.002 | 0.277 |
| **Family** | | | | | | | | | | |
| Ruminococcaceae | 0.210 | 0.237 | 0.195 | 0.222 | 0.136 | 0.208 | 0.241 | 0.213 | 0.012 | 0.682 |
| ClostridiaUCG014 | 0.162 | 0.236 | 0.143 | 0.114 | 0.157 | 0.217 | 0.196 | 0.224 | 0.015 | 0.270 |
| Lachnospiraceae | 0.075 | 0.058 | 0.140 | 0.106 | 0.186 | 0.113 | 0.087 | 0.079 | 0.015 | 0.210 |
| Eubacterium_coprostanoligenes group | 0.172 | 0.115 | 0.095 | 0.137 | 0.086 | 0.056 | 0.071 | 0.060 | 0.014 | 0.860 |
| RF39 | 0.085 | 0.126 | 0.116 | 0.081 | 0.047 | 0.095 | 0.113 | 0.099 | 0.009 | 0.490 |
| Oscillospiraceae | 0.063 | 0.065 | 0.101 | 0.076 | 0.013 | 0.073 | 0.069 | 0.065 | 0.009 | 0.090 |
| Enterobacteriaceae | 0.002 | 0.0002 | 0.003 | 0.014 | 0.181* | 0.003 | 0.001 | 0.001 | 0.022 | 0.009 |
| ClostridiavadinBB60group | 0.028 | 0.033 | 0.026 | 0.022 | 0.006 | 0.036 | 0.023 | 0.023 | 0.003 | 0.170 |
| UCG010 | 0.023 | 0.030 | 0.034 | 0.024 | 0.003 | 0.018 | 0.018 | 0.035 | 0.004 | 0.200 |
| Lactobacillaceae | 0.035 | 0.004 | 0.025 | 0.030 | 0.019 | 0.026 | 0.006 | 0.024 | 0.004 | 0.470 |
| Akkermansiaceae | 0.034 | 0.003 | 0.005 | 0.025 | 0.003 | 0.019 | 0.045 | 0.031 | 0.006 | 0.860 |
| Christensenellaceae | 0.025 | 0.019 | 0.017 | 0.016 | 0.007 | 0.032 | 0.021 | 0.018 | 0.003 | 0.090 |
| Erysipelatoclostridiaceae | 0.009 | 0.010 | 0.010 | 0.027 | 0.036 | 0.014 | 0.008 | 0.008 | 0.004 | 0.070 |
| Erysipelotrichaceae | 0.008 | 0.005 | 0.010 | 0.011 | 0.027 | 0.011 | 0.014 | 0.020 | 0.003 | 0.170 |
| Streptococcaceae | 0.004 | 0.001 | 0.022 | 0.012 | 0.001 | 0.006 | 0.017 | 0.041 | 0.005 | 0.120 |
| Butyricicoccaceae | 0.012 | 0.006 | 0.007 | 0.015 | 0.011 | 0.006 | 0.021 | 0.019 | 0.002 | 0.090 |
| Monoglobaceae | 0.010 | 0.017 | 0.009 | 0.011 | 0.009 | 0.010 | 0.017 | 0.010 | 0.001 | 0.930 |
| Anaerovoracaceae | 0.006 | 0.005 | 0.004 | 0.003 | 0.020 | 0.010 | 0.006 | 0.003 | 0.002 | 0.180 |
| Unclassified | 0.008 | 0.003 | 0.010 | 0.008 | 0.007 | 0.011 | 0.004 | 0.006 | 0.001 | 0.810 |
| Eggerthellaceae | 0.006 | 0.005 | 0.010 | 0.010 | 0.004 | 0.011 | 0.004 | 0.005 | 0.001 | 0.460 |
| **Genera** | | | | | | | | | | |
| Clostridia UCG014 | 0.162 | 0.236 | 0.143 | 0.114 | 0.157 | 0.217 | 0.196 | 0.224 | 0.015 | 0.274 |
| *[Eubacterium] coprostanoligenes* group | 0.172 | 0.115 | 0.095 | 0.137 | 0.086 | 0.056 | 0.071 | 0.060 | 0.014 | 0.863 |
| RF39 | 0.085 | 0.126 | 0.116 | 0.081 | 0.047 | 0.095 | 0.113 | 0.099 | 0.009 | 0.487 |
| *Subdoligranulum* | 0.063 | 0.081 | 0.045 | 0.075 | 0.047 | 0.085 | 0.091 | 0.082 | 0.006 | 0.795 |
| *Ruminococcus torques* group | 0.030 | 0.029 | 0.095 | 0.045 | 0.072 | 0.064 | 0.040 | 0.030 | 0.009 | 0.307 |
| *Faecalibacterium* | 0.068 | 0.056 | 0.039 | 0.059 | 0.026 | 0.022 | 0.063 | 0.048 | 0.006 | 0.862 |
| *Escherichia-Shigella* | 0.002 | 0.0002 | 0.003 | 0.014 | 0.181* | 0.003 | 0.001 | 0.001 | 0.022 | 0.009 |
| Clostridia vadin BB60 group | 0.028 | 0.033 | 0.026 | 0.022 | 0.006 | 0.036 | 0.023 | 0.023 | 0.003 | 0.171 |
| UCG010 | 0.023 | 0.030 | 0.034 | 0.024 | 0.003 | 0.018 | 0.018 | 0.035 | 0.004 | 0.204 |
| *Lactobacillus* | 0.035 | 0.004 | 0.025 | 0.030 | 0.019 | 0.026 | 0.006 | 0.024 | 0.004 | 0.471 |
| *Akkermansia* | 0.034 | 0.003 | 0.005 | 0.025 | 0.003 | 0.019 | 0.045 | 0.031 | 0.006 | 0.862 |
| Christensenellaceae R7 group | 0.025 | 0.019 | 0.017 | 0.016 | 0.006 | 0.032 | 0.021 | 0.018 | 0.003 | 0.113 |
| *Erysipelatoclostridium* | 0.009 | 0.010 | 0.010 | 0.027 | 0.036 | 0.014 | 0.008 | 0.008 | 0.004 | 0.073 |
| *Lachnoclostridium* | 0.010 | 0.011 | 0.012 | 0.016 | 0.016 | 0.020 | 0.010 | 0.013 | 0.001 | 0.559 |
| Incertae sedis | 0.015 | 0.012 | 0.017 | 0.009 | 0.013 | 0.016 | 0.014 | 0.012 | 0.001 | 0.943 |
| *Streptococcus* | 0.004 | 0.001 | 0.022 | 0.012 | 0.001 | 0.006 | 0.017 | 0.041 | 0.005 | 0.118 |
| *Monoglobus* | 0.010 | 0.017 | 0.009 | 0.011 | 0.009 | 0.010 | 0.017 | 0.010 | 0.001 | 0.926 |
| NK4A214 group | 0.010 | 0.007 | 0.015 | 0.011 | 0.005 | 0.017 | 0.007 | 0.008 | 0.002 | 0.595 |
| Erysipelotrichaceae | 0.008 | 0.004 | 0.009 | 0.009 | 0.009 | 0.010 | 0.003 | 0.020 | 0.002 | 0.438 |
| *Negativibacillus* | 0.010 | 0.006 | 0.011 | 0.014 | 0.005 | 0.005 | 0.006 | 0.015 | 0.001 | 0.432 |

^1^BSFL – basal diet with 100% black soldier fly (*Hermetia illucens*) larval fat; ^2^SO – basal diet with 100% soybean oil; ^3^RO – basal diet with 100% rapeseed oil; ^4^PO – basal diet with 100% palm oil; ^5^PKFD – basal diet with 100% palm kernel fat distillate; ^6^PF – basal diet with 100% poultry fat; ^7^PLbasal diet with 100% pig lard; ^8^BT – basal diet with 100% beef tallow; ^9^SEM – standard error of the mean
