# Supplementary material for: Cecal microbiota of broilers responds similarly to black soldier fly larvae fat and conventional dietary fat sources
Source: PLoS One. 2025 Nov 25;20(11):e0336523. doi: 10.1371/journal.pone.0336523 (PMC12646438; doi:10.1371/journal.pone.0336523)
Supplement: S2 Table — (DOCX) [file pone.0336523.s002.docx]

**S2 Table.** Biomarker identification in broiler chicken ceca via LEfSe analysis (3 groups)

|  | Feature | Group | ef_lda | P value |
| --- | --- | --- | --- | --- |
| marker1 | d__Bacteria | ^1^AF | 4.705 | < 0.001 |
| marker2 | d__Bacteria\|p__Firmicutes\|c__Clostridia\|o__Clostridia_UCG-014\|f__Clostridia_UCG-014\|g__Clostridia_UCG-014\|g__Clostridia_UCG-014_s__uncultured_bacterium | AF | 4.634 | 0.012 |
| marker3 | d__Bacteria\|p__Firmicutes\|c__Clostridia\|o__Clostridia_vadinBB60_group\|f__Clostridia_vadinBB60_group\|g__Clostridia_vadinBB60_group\|g__Clostridia_vadinBB60_group_s__unidentified | AF | 2.196 | 0.014 |
| marker4 | d__Bacteria\|p__Proteobacteria\|c__Alphaproteobacteria\|o__Sphingomonadales\|f__Sphingomonadaceae | AF | 2.148 | 0.009 |
| marker5 | d__Bacteria\|p__Proteobacteria\|c__Alphaproteobacteria\|o__Sphingomonadales\|f__Sphingomonadaceae\|g__Sphingomonas | AF | 2.148 | 0.009 |
| marker6 | d__Bacteria\|p__Proteobacteria\|c__Alphaproteobacteria\|o__Sphingomonadales | AF | 2.148 | 0.009 |
| marker7 | d__Bacteria\|p__Firmicutes\|c__Clostridia\|o__Peptococcales\|f__Peptococcaceae\|f__Peptococcaceae_g__uncultured\|fPeptococcaceae_g_uncultured | ^2^BSFL | 2.470 | 0.019 |
| marker8 | d__Bacteria\|p__Firmicutes\|c__Bacilli\|o__Erysipelotrichales\|f__Erysipelatoclostridiaceae\|g__Erysipelatoclostridium\|g__Erysipelatoclostridium_s__Clostridiales_bacterium | ^3^Plant oil | 2.820 | 0.047 |
| marker9 | d__Bacteria\|p__Firmicutes\|c__Clostridia\|o__Oscillospirales\|f__Oscillospiraceae\|g__UCG-005\|g__UCG-005_s__uncultured_bacterium | Plant oil | 2.614 | 0.038 |
| marker10 | d__Bacteria\|p__Firmicutes\|c__Clostridia\|o__Lachnospirales\|f__Lachnospiraceae\|gSellimonas\|g_Sellimonas | Plant oil | 2.495 | 0.013 |
| marker11 | d__Bacteria\|p__Firmicutes\|c__Clostridia\|o__Lachnospirales\|f__Lachnospiraceae\|g__Anaerostipes\|s__Anaerostipes_butyraticus | Plant oil | 2.204 | 0.044 |

^1^AF- Animal Fat (PF–100% poultry fat; PL –100% pig lard; BT –100% beef tallow); ^2^BSFL – basal diet with 100% black soldier fly (*Hermetia illucens*) larval fat; ^3^plant oil (SO –100% soybean oil; RO –100% rapeseed oil; PO –100% palm oil; PKFD –100% palm kernel fat distillate); ef_lda – effect size from linear discriminant analysis.
