# Supplementary material for: Cecal microbiota of broilers responds similarly to black soldier fly larvae fat and conventional dietary fat sources
Source: PLoS One. 2025 Nov 25;20(11):e0336523. doi: 10.1371/journal.pone.0336523 (PMC12646438; doi:10.1371/journal.pone.0336523)
Supplement: S3 Table — (DOCX) [file pone.0336523.s003.docx]

**S3 Table.** Biomarker identification in broiler chicken ceca via LEfSe analysis (8 groups)

|  | Feature | Group | ef_lda | P value |
| --- | --- | --- | --- | --- |
| marker1 | d__Bacteria\|p__Proteobacteria\|c__Gammaproteobacteria\|o__Enterobacterales\|f__Enterobacteriaceae | PKFD | 4.61 | 0.010 |
| marker2 | d__Bacteria\|p__Proteobacteria\|c__Gammaproteobacteria | PKFD | 4.61 | 0.015 |
| marker3 | d__Bacteria\|p__Proteobacteria\|c__Gammaproteobacteria\|o__Enterobacterales | PKFD | 4.61 | 0.010 |
| marker4 | d__Bacteria\|p__Proteobacteria | PKFD | 4.61 | 0.015 |
| marker5 | d__Bacteria\|p__Proteobacteria\|c__Gammaproteobacteria\|o__Enterobacterales\|f__Enterobacteriaceae\|g__Escherichia-Shigella\|s__Escherichia_coli | PKFD | 4.61 | 0.010 |
| marker6 | d__Bacteria | BSFL | 4.54 | <0.001 |
| marker7 | d__Bacteria\|pFirmicutes\|c_Clostridia\|oOscillospirales\|f_Oscillospiraceae\|fOscillospiraceae\|f_Oscillospiraceae | PO | 4.15 | 0.040 |
| marker8 | d__Bacteria\|p__Firmicutes\|c__Clostridia\|o__Oscillospirales\|f__Oscillospiraceae\|f__Oscillospiraceae_g__uncultured\|fOscillospiraceae_g_uncultured | RO | 3.98 | 0.041 |
| marker9 | d__Bacteria\|p__Firmicutes\|c__Clostridia\|o__Lachnospirales\|f_Lachnospiraceae\|fLachnospiraceae | PKFD | 3.92 | 0.043 |
| marker10 | d__Bacteria\|p__Firmicutes\|c__Clostridia\|o__Oscillospirales\|f__Butyricicoccaceae\|g__Butyricicoccus\|s__Butyricicoccus_pullicaecorum | BT | 3.78 | 0.039 |
| marker11 | d__Bacteria\|p__Firmicutes\|c__Clostridia\|o__Oscillospirales\|f__Butyricicoccaceae\|g__Butyricicoccus\|g__Butyricicoccus_s__uncultured_bacterium | PL | 3.75 | 0.034 |
| marker12 | d__Bacteria\|p__Firmicutes\|c__Clostridia\|o__Oscillospirales\|f__Ruminococcaceae\|f__Ruminococcaceae_g__uncultured\|s__human_gut | RO | 3.64 | 0.017 |
| marker13 | d__Bacteria\|p__Firmicutes\|c__Bacilli\|o__Erysipelotrichales\|f__Erysipelotrichaceae\|g__Turicibacter | PKFD | 3.61 | 0.002 |
| marker14 | d__Bacteria\|p__Firmicutes\|c__Clostridia\|o__Oscillospirales\|f__Ruminococcaceae\|g__Ruminococcus\|g__Ruminococcus_s__Ruminococcus_sp. | PL | 3.51 | 0.038 |
| marker15 | d__Bacteria\|p__Firmicutes\|c__Clostridia\|o__Lachnospirales\|f__Lachnospiraceae\|g__Eisenbergiella\|g__Eisenbergiella_s__uncultured_bacterium | PKFD | 3.41 | 0.036 |
| marker16 | d__Bacteria\|p__Firmicutes\|c__Clostridia\|o__Lachnospirales\|f__Lachnospiraceae\|g__Lachnospiraceae_UCG-010\|g__Lachnospiraceae_UCG-010_s__uncultured_bacterium | PL | 3.00 | 0.035 |
| marker17 | d__Bacteria\|p__Bacteroidota\|c__Bacteroidia\|o__Bacteroidales\|f__Rikenellaceae\|g__Alistipes\|s__Alistipes_sp. | BSFL | 2.94 | 0.023 |
| marker18 | d__Bacteria\|p__Bacteroidota\|c__Bacteroidia\|o__Bacteroidales\|f__Rikenellaceae | BSFL | 2.94 | 0.025 |
| marker19 | d__Bacteria\|p__Bacteroidota\|c__Bacteroidia\|o__Bacteroidales\|f__Rikenellaceae\|g__Alistipes | BSFL | 2.94 | 0.025 |
| marker20 | d__Bacteria\|p__Firmicutes\|c__Clostridia\|o__uncultured\|o__uncultured_f__uncultured\|o__uncultured_f__uncultured_g__uncultured\|o__uncultured_f__uncultured_g__uncultured_s__uncultured_bacterium | PKFD | 2.94 | 0.034 |
| marker21 | d__Bacteria\|p__Firmicutes\|c__Clostridia\|o__Lachnospirales\|f__Defluviitaleaceae\|g__Defluviitaleaceae_UCG-011\|g__Defluviitaleaceae_UCG-011_s__uncultured_bacterium | PKFD | 2.93 | 0.035 |
| marker22 | d__Bacteria\|p__Firmicutes\|c__Clostridia\|o__Lachnospirales\|f__Defluviitaleaceae | PKFD | 2.93 | 0.035 |
| marker23 | d__Bacteria\|p__Firmicutes\|c__Clostridia\|o__Lachnospirales\|f__Defluviitaleaceae\|g__Defluviitaleaceae_UCG-011 | PKFD | 2.93 | 0.035 |
| marker24 | d__Bacteria\|p__Firmicutes\|c__Clostridia\|o__Lachnospirales\|f__Lachnospiraceae\|gTuzzerella\|g_Tuzzerella | PL | 2.91 | 0.044 |
| marker25 | d__Bacteria\|p__Firmicutes\|c__Clostridia\|o__Oscillospirales\|f__Butyricicoccaceae\|g__Butyricicoccus\|g__Butyricicoccus_s__uncultured_Firmicutes | PKFD | 2.84 | 0.004 |
| marker26 | d__Bacteria\|p__Firmicutes\|c__Clostridia\|o__Oscillospirales\|f__Oscillospiraceae\|g__UCG-005\|g__UCG-005_s__uncultured_bacterium | SO | 2.79 | 0.015 |
| marker27 | d__Bacteria\|p__Firmicutes\|c__Clostridia\|o__Lachnospirales\|f__Lachnospiraceae\|g__Blautia\|s__Blautia_hydrogenotrophica | PKFD | 2.71 | 0.042 |
| marker28 | d__Bacteria\|p__Firmicutes\|c__Clostridia\|o__Lachnospirales\|f__Lachnospiraceae\|gSellimonas\|g_Sellimonas | PKFD | 2.63 | 0.010 |
| marker29 | d__Bacteria\|p__Firmicutes\|c__Clostridia\|o__Oscillospirales\|f__Ruminococcaceae\|g__Angelakisella\|g__Angelakisella_s__Ruminococcus_sp. | SO | 2.51 | 0.016 |
| marker30 | d__Bacteria\|p__Firmicutes\|c__Clostridia\|o__Oscillospirales\|f__Ruminococcaceae\|g__Angelakisella | SO | 2.51 | 0.016 |
| marker31 | d__Bacteria\|p__Firmicutes\|c__Clostridia\|o__Peptococcales\|f__Peptococcaceae\|f__Peptococcaceae_g__uncultured\|fPeptococcaceae_g_uncultured | BSFL | 2.48 | 0.003 |
| marker32 | d__Bacteria\|p__Proteobacteria\|c__Gammaproteobacteria\|o__Enterobacterales\|f__Enterobacteriaceae\|gEscherichia-Shigella\|g_Escherichia-Shigella | PKFD | 2.47 | <0.001 |
| marker33 | d__Bacteria\|p__Firmicutes\|c__Clostridia\|o__Lachnospirales\|f__Lachnospiraceae\|g__CAG-56 | PKFD | 2.47 | 0.002 |
| marker34 | d__Bacteria\|p__Firmicutes\|c__Clostridia\|o__Oscillospirales\|o__Oscillospirales_f__uncultured\|o__Oscillospirales_f__uncultured_g__uncultured\|o__Oscillospirales_f__uncultured_g__uncultured_s__uncultured_bacterium | PL | 2.39 | 0.026 |
| marker35 | d__Bacteria\|p__Proteobacteria\|c__Alphaproteobacteria | PL | 2.37 | 0.042 |
| marker36 | d__Bacteria\|p__Firmicutes\|c__Bacilli\|o__Erysipelotrichales\|f__Erysipelotrichaceae\|f__Erysipelotrichaceae_g__uncultured\|f__Erysipelotrichaceae_g__uncultured_s__Clostridiales_bacterium | PKFD | 2.37 | 0.001 |
| marker37 | d__Bacteria\|p__Firmicutes\|c__Bacilli\|o__Erysipelotrichales\|f__Erysipelotrichaceae\|f__Erysipelotrichaceae_g__uncultured | PKFD | 2.37 | 0.001 |
| marker38 | d__Bacteria\|p__Firmicutes\|c__Clostridia\|o__Lachnospirales\|f__Lachnospiraceae\|g__Anaerostipes\|s__Anaerostipes_butyraticus | PKFD | 2.31 | 0.008 |
| marker39 | d__Bacteria\|p__Proteobacteria\|c__Gammaproteobacteria\|o__Pseudomonadales | PL | 2.26 | 0.018 |
| marker40 | d__Bacteria\|p__Firmicutes\|c__Clostridia\|o__Lachnospirales\|f__Lachnospiraceae\|gMarvinbryantia\|g_Marvinbryantia | PKFD | 2.25 | 0.026 |
| marker41 | d__Bacteria\|p__Firmicutes\|c__Clostridia\|oOscillospirales\|f[Eubacterium]_coprostanoligenes_group\|g__[Eubacterium]_coprostanoligenes_group\|g__[Eubacterium]_coprostanoligenes_group_s__unidentified | BSFL | 2.23 | 0.018 |
| marker42 | d__Bacteria\|p__Firmicutes\|c__Clostridia\|o__Clostridia_UCG-014\|f__Clostridia_UCG-014\|g__Clostridia_UCG-014\|s__uncultured_Ruminococcaceae | PKFD | 2.23 | 0.043 |
| marker43 | d__Bacteria\|p__Firmicutes\|c__Clostridia\|o__Oscillospirales\|o__Oscillospirales_f__uncultured\|o__Oscillospirales_f__uncultured_g__uncultured\|o__Oscillospirales_f_uncultured_guncultured | PKFD | 2.17 | 0.038 |
| marker44 | d__Bacteria\|p__Firmicutes\|c__Clostridia\|o__Lachnospirales\|f__Lachnospiraceae\|g__CHKCI001\|g__CHKCI001_s__uncultured_organism | PKFD | 2.14 | 0.006 |
| marker45 | d__Bacteria\|p__Proteobacteria\|c__Alphaproteobacteria\|o__Sphingomonadales\|f__Sphingomonadaceae | PL | 2.14 | 0.025 |
| marker46 | d__Bacteria\|p__Proteobacteria\|c__Alphaproteobacteria\|o__Sphingomonadales\|f__Sphingomonadaceae\|g__Sphingomonas | PL | 2.14 | 0.025 |
| marker47 | d__Bacteria\|p__Proteobacteria\|c__Alphaproteobacteria\|o__Sphingomonadales | PL | 2.14 | 0.025 |
| marker48 | d__Bacteria\|p__Firmicutes\|c__Clostridia\|o__Eubacteriales\|f__Anaerofustaceae\|g__Anaerofustis\|s__Anaerofustis_sp. | PKFD | 2.14 | 0.044 |
| marker49 | d__Bacteria\|p__Firmicutes\|c__Clostridia\|o__Eubacteriales\|f__Anaerofustaceae | PKFD | 2.14 | 0.044 |
| marker50 | d__Bacteria\|p__Firmicutes\|c__Clostridia\|o__Eubacteriales\|f__Anaerofustaceae\|g__Anaerofustis | PKFD | 2.14 | 0.044 |
| marker51 | d__Bacteria\|p__Firmicutes\|c__Clostridia\|o__Eubacteriales | PKFD | 2.13 | 0.044 |
| marker52 | d__Bacteria\|p__Proteobacteria\|c__Alphaproteobacteria\|o__Rhodospirillales\|o__Rhodospirillales_f__uncultured\|o__Rhodospirillales_f__uncultured_g__uncultured | BSFL | 2.05 | 0.048 |
| marker53 | d__Bacteria\|p__Proteobacteria\|c__Alphaproteobacteria\|o__Rhodospirillales | BSFL | 2.05 | 0.048 |
| marker54 | d__Bacteria\|p__Firmicutes\|c__Clostridia\|o__Clostridia_vadinBB60_group\|f__Clostridia_vadinBB60_group\|gClostridia_vadinBB60_group\|g_Clostridia_vadinBB60_group | BSFL | 2.04 | 0.032 |
| marker55 | d__Bacteria\|p__Proteobacteria\|c__Gammaproteobacteria\|o__Pseudomonadales\|f__Moraxellaceae | PL | 2.03 | 0.024 |
| marker56 | k_Bacteria\|p_Firmicutes\|c_Clostridia\|o_Lachnospirales\|f_Lachnospiraceae\|g_Lachnospiraceae_FE2018_group\|s_uncultured bacterium | PKFD | 2.05 | 0.001 |

BSFL – basal diet with 100% black soldier fly (*Hermetia illucens*) larval fat; SO – basal diet with 100% soybean oil; RO – basal diet with 100% rapeseed oil; PO – basal diet with 100% palm oil; PKFD – basal diet with 100% palm kernel fat distillate; PL – basal diet with 100% pig lard; BT – basal diet with 100% beef tallow; ef_lda – effect size from linear discriminant analysis.
